# Genome Sequence of the Poly-3-Hydroxybutyrate Producer *Clostridium acetireducens* DSM 10703

**DOI:** 10.1128/genomeA.01399-16

**Published:** 2016-12-15

**Authors:** Sebastian Flüchter, Anja Poehlein, Bettina Schiel-Bengelsdorf, Rolf Daniel, Peter Dürre

**Affiliations:** a Institut für Mikrobiologie und Biotechnologie, Universität Ulm, Ulm, Germany; b Genomic and Applied Microbiology and Göttingen Genomics Laboratory, Georg-August University, Göttingen, Germany

## Abstract

Here, we report the genome sequence of *Clostridium acetireducens* (DSM 10703^T^), a strictly anaerobic bacterium capable of fermenting acetate and leucine to butyrate, isovalerate, and poly-3-hydroxybutyrate. The draft genome consists of a circular chromosome with a size of 2.4 Mb and harbors 2,239 predicted protein-encoding genes.

*Clostridium acetireducens* DSM 10703 was first isolated in 1996 from a bioreactor at a potato starch factory in de Krim, Netherlands (1). It was described as a nonmotile, Gram-positive, anaerobic, rod-shaped organism with the ability to oxidize various amino acids, including alanine, leucine, isoleucine, valine, serine, and threonine, together with acetate as an electron acceptor. Later, the organism was further characterized, and it was reported that in the presence of leucine and acetate *C. acetireducens* produces isovalerate, butyrate, and poly-3-hydroxybutyrate (2). 

Genomic DNA was isolated using a MasterPure Gram^+^ DNA purification kit (Biozym, Hessisch Oldendorf, Germany). Extracted DNA was used to generate Illumina shotgun paired-end sequencing libraries, which were sequenced with a MiSeq instrument and the MiSeq reagent kit version 3, as recommended by the manufacturer (Illumina, San Diego, CA, USA). Quality filtering using Trimmomatic version 0.32 (3) resulted in 2,596,354 paired-end reads (301 bp). The assembly was performed with the SPAdes genome assembler software version 3.8.0 (4) and resulted in 85 contigs (>500 bp) with an average coverage of 229-fold. QualiMap version 2.1 (5) was used to validate the assembly and to determine the read coverage. The draft genome of *C. acetireducens* DSM 10703 consists of a single chromosome (2.4 Mb) with an overall GC content of 26.75%. Automatic gene prediction and identification of rRNA and tRNA genes was performed using the software tool Prokka (6). The draft genome harbored eight rRNA genes, 69 tRNA genes, 1,715 protein-encoding genes with function prediction, and 524 genes coding for hypothetical proteins.

Genome analysis revealed the presence of a gene cluster that included *phaJEC*, which is required for poly-3-hydroxybutyrate production. BLAST analysis indicated high similarity to *phaEC* genes from cyanobacteria, which belong to class III polyhydroxyalkanoate (PHA) synthases (7). A high similarity was also detected to *phaEC* gene clusters present in *Clostridium homopropionicum* DSM 5847 (8) and to *phaJEC* of *C. lundense* DSM 17049 (downloaded at IMG [9]) and *C. tetanomorphum* DSM 665 (10). The *phaEC* (CLOACE_21150 and CLOACE_21140) gene cluster encodes a PHA synthase class III (11), and *phaJ* encodes for an (R)-enoyl-CoA hydratase (CLOACE_21160), which exhibits 48% similarity to PhaJ_Hm_ of *Haloferax mediterranei* R-4 ATCC 33500 (12). PhaJ_Hm_ converts crotonyl-CoA to (R)-3-hydroxybutyryl-CoA, the precursor for poly-3-hydroxybutyrate synthesis (13).

## Accession number(s).

This whole-genome shotgun project has been deposited at DDBJ/ENA/GenBank under the accession number LZFO00000000. The version described in this paper is the first version, LZFO01000000.
